# Mobile application e-grocery retail adoption challenges and coping strategies: a South African small and medium enterprises’ perspective

**DOI:** 10.1007/s10660-023-09698-1

**Published:** 2023-04-25

**Authors:** Marcia Mkansi, Aaron Luntala Nsakanda

**Affiliations:** 1 Sprott School of Business, Carleton University, 1125 Colonel By Drive, Ottawa, Ontario Canada; 2 Department of Operations Management, University of South Africa, Preller Street, Pretoria, 0002 South Africa

**Keywords:** Mobile applications, E-grocery retailers, E-business, SME, m-commerce

## Abstract

This paper explores how small and medium-sized e-grocery mobile application retailers evolving within the geographical context of South Africa and operating in the urban, township, and rural areas respond to theoretically and emerging field-based e-business and e-grocery adoption challenges, respectively. The study used semi-structured qualitative interviews to explore the coping strategies of e-grocery mobile application retailers to mitigate technological, organizational, and environmental (TOE) adoption challenges. The significance of small grocery adoption strategies related to context informs e-grocery adoption from the evidence generated in other small e-grocers and for the superior grade of TOE (or theoretical) knowledge sought from the inevitable evolving mobile application and digital grocery markets. The findings reveal that specialist skills and unified team production are crucial conduits for lowering the TOE barriers to e-business and e-grocery adoption. They also reveal the interconnected resource orchestration, shared value, and social inclusion strategies used to mitigate various e-business and e-grocery challenges.

**Supplementary Information:**

The online version contains supplementary material available at 10.1007/s10660-023-09698-1.

## Introduction

The high penetration of mobile devices has fueled the development of mobile application-based business models, or mobile commerce (m-commerce) [1, 2], which has transformed the trading and shopping channels across different sectors, ranging from grocery, banking, apparel, and travel to music [3]. According to earlier studies [1, 2], 58.9% of e-commerce sales were attributed to mobile commerce in 2017, with the expectation to reach 72.9% in 2021. In 2018, over 3.5 billion people were connected to mobile phones [4–6]. In Africa, 67% of the population (approximately 1.13 billion people) had mobile phones in 2015 [7], and the figures are currently in the range of 46% in Southern Africa and 49% in North Africa [6]. The exponential growth of mobile connections represents market opportunities for mobile application-based business models.

Although mobile application-based businesses are on the rise globally, e-grocery adoption is still relatively low across businesses of all sizes. The total $189.81bn global online grocery sales [8] constitute 0.00005% of the 2019 global e-commerce retail sales of $3.5 trillion [9]. In 2018, for example, Africa and Asia–Pacific accounted for only 3.4% of e-grocery adoption [10]. Comparatively, in 2018, 35.4% of the market share was held by the top ten global grocery markets of China, the United States (US), Japan, the United Kingdom (UK), South Korea, France, Australia, Germany, Canada, and Spain [11]. Most of the market share mentioned above is held by the top ten online global grocery retailers: Amazon, Wal-Mart Stores, Alibaba Group, Tesco plc, Casino Group, Costco, Target, Leclerc, Auchan Group, and Sainsbury’s [12]. SMEs are still lagging due to unresolved general e-business adoption challenges [13, 14]. However, even for large organizations, the challenges are amplified and worse in e-grocery retail [12, 15] than in other sectors.

Recently, e-business adoption by SMEs has been studied extensively using various theoretical lenses that include, but are not limited to, the resource-based theory (RBT), Roger’s diffusion of innovation theory (DOI), and the technology–organization–environment (TOE) framework [13, 14]. However, critiques of the theories discussed in-depth by Mkansi [16] paint a masterly depiction of inconclusiveness and unsuitability for SMEs’ e-business adoption decisions. Despite the limitations, TOE’s theoretical framework is highly recommended because it embodies the key constructs that correlate with the source of challenges and the operational context of small and medium-sized businesses. Recent reviews of studies on SMEs’ e-business adoption [17–20] reiterate that TOE is an advanced lens to explain SMEs’ e-business adoption. Deng et al. [17] used TOE to explore SMEs’ critical determinants of electronic market adoption. Arslan et al. [18] investigated the TOE factors associated with information and communication technologies (ICT) within the context of both small and large manufacturing firms. Olaitan and Flowerday [19] applied TOE to assess successful IT governance in small and medium enterprises. Maduku et al. [20] used TOE to understand the mobile marketing intention of SMEs in South Africa (SA). Similar to the scholars mentioned above, this study adopted TOE as a lens for exploring e-grocery adoption and strategies. In order to complement some of TOE's weaknesses, this study added Roger’s key attributes, such as perceived advantage, ease of use, compatibility, trialability, perceived importance of compliance, and perceived risks, in the exploration of e-grocery adoption. TOE posits that internal and external technology infrastructure; the scope, size, organizational structure; and environmental contexts beyond the control of the organizations are significant constructs for diagnosing e-business adoption [13, 21].

While the latter studies [17, 18] provided a great deal of e-business adoption, there needs to be more research that explores how the TOE presents adoption challenges to small and medium-sized e-grocery retailers. Furthermore, research on how SMEs cope with adoption challenges and any other emerging challenges across the TOE contexts still needs to be explored. The exception is Mkansi’s [16] study of e-business adoption costs and strategies for small businesses. However, Mkansi’s [16] study is limited to clothing retail, which differs from the e-grocery sector, where options related to last-mile delivery stand to be resolved.

The participation of SMEs in the e-grocery sector is critical, considering that they are the engine of economic growth [17], are linked to 80% of global economic growth [22], and make a 90% contribution to the African continent’s economy. Therefore, it needs to be asked how research can contribute to the development of SMEs and reposition those in the e-grocery sector to play a meaningful role in the transformation of developing economies and allow access to the global online grocery market that is expected to grow from $189.81 billion in 2019 [8], up to $975.16 billion in 2027 [23].

This study argues that an understanding of the e-grocery adoption challenges from the perspective of the TOE is a necessary action toward transforming and repositioning SMEs. It repositions the TOE framework beyond the factors influencing the adoption to encompass the explanatory powers of challenges and mitigating strategies. The latter argument fosters a renewed approach that facilitates knowledge about and a greater understanding of adoption, seen in line with the e-grocery challenges. The study focuses on small and medium-sized e-grocery mobile application retailers evolving within the geographical context of South Africa as they offer unique opportunities to exhibit the heterogeneity and distinctive attributes that can complement the review and understanding of operations not only in urban and rural areas but also in the township areas. An e-grocery mobile application is a platform that uses a software application (app) to browse and order grocery items, stream live e-grocery orders to various stakeholders, and manage the complexities associated with e-grocery delivery operations [15]. Depending on the underlying business model, the platform can serve as an aggregator, a marketplace, a single store, or a supermarket chain. The current study sought to explore the following research questions:

- RQ1 What specific challenges are experienced by small e-grocery mobile application retailers?
- RQ2 How do small e-grocery mobile application retailers cope with the challenges they experience?
- RQ3 How do the challenges and strategies align with the TOE’s adoption framework?

We address the research questions by first developing a comprehensive list of respectively e-business and e-grocery adoption challenges from the literature and situating them within the apparatus of TOE theory. This list sets out the theoretical condensed double-layered e-business/e-grocery adoption challenges, which comprise the general e-business adoption challenges augmented with the e-grocery adoption context challenges. The approach is central to exploring the relationships between how the double-edged e-business and e-grocery challenges occur and relate to the TOE environment and SME structure, which is highly characterized by dynamic mobile apps, and the complex logistics peculiar to townships and rural settings. Next, we asked practitioners whether they had experienced these adoption challenges and their coping strategies to overcome them. We also asked practitioners to report any other field-based emerging challenges they had encountered and how they handled them. The practitioners’ inputs recognized Pollard and Morale’s [24] quest to inform theory from practice, which is fundamental to capturing the dynamic and rapidly changing environment of e-business/e-grocery adoption as it manifests within the unique context of the SMEs.

The remainder of the paper is structured as follows. The next section reviews the e-grocery adoption challenges to produce a comprehensive and complementary view of the adoption challenges faced by small and medium-sized e-grocery retailers. Drawing on TOE, the comprehensive view is consolidated to address the wide variety of e-grocery adoption context challenges. Section 3 describes the qualitative methods used, followed by the findings and their discussion in Sects. 4 and 5, respectively. Concluding remarks are presented in Sect. 6.

## Literature review

The adoption of e-business does not present homogeneous challenges for large businesses and SMEs, as well as across all sectors of activity. This section briefly discusses the general challenges associated with adopting e-business that small businesses face in any industry. Then, it focuses on the e-grocery adoption challenges to produce a comprehensive and complementary view of the adoption challenges faced by small grocery e-retailers. Finally, drawing on TOE theoretical framework, the comprehensive view is consolidated to address the wide variety of e-grocery industry context adoption challenges.

### E-business adoption challenges

Several scholars documented SME barriers to e-business adoption [25–30]. Zaide [25] highlighted issues beyond the SMEs’ control, such as immature logistics and telecommunication infrastructure, low-income penetration, and credit card penetration. The Organization for Economic Co-operation and Development (OECD) [26] pointed to the limited capabilities of SMEs to provide the secure payment infrastructure necessary to foster trust and privacy concerns. The International Trade Centre (ITC) [26] attested that network facilities, to a large extent, are a significant source of direct costs and barriers to SMEs’ e-business adoption. Ongori and Migiro [28] pointed towards organizational challenges such as limited finances, skills, and knowledge and the lack of expensive financial resources to deal with legal issues, maintenance, and upgrades. Hove and Karimov [29] revealed the risks and the high cost of addressing delivery and payment security. Cloete et al. [30] argued that SMEs require time and information support. Kapurubandara [31] discussed a roadmap of barriers that hinder SMEs in developing countries at the different stages of e-business adoption. Accordingly, this paper outlines the issues that need to address before moving from one stage to the next. For their part, Shi et al. [32] discussed models that provide practical guidance to SMEs on when to adopt e-business while facing uncertain operating costs. However, this work does not emphasize SME strategies to address barriers to e-business adoption.

Although most e-business adoption challenges relevant to SMEs are presented outside the TOE theoretical framework, most are constructed around the interplay of technology, organization, and environmental stances. Notably, the e-business adoption challenges presented by the scholars mentioned above are situated outside the context of the e-grocery sector. Thus, this limits the discoveries that accurately reflect the proper context of SMEs in the e-grocery sectors. Put differently, technology adoption challenges require explicit heterogeneous peculiarities. The heterogeneous nature of SMEs motivates an exploration of the double-layered adoption challenges that emanate from the e-business domain and the sub-category of the sectoral e-grocery context. Therefore, the context offers renewed theoretical explanatory powers that account for e-business/e-grocery adoption that reflects practice.

### E-grocery adoption challenges

**Fig. 1:**
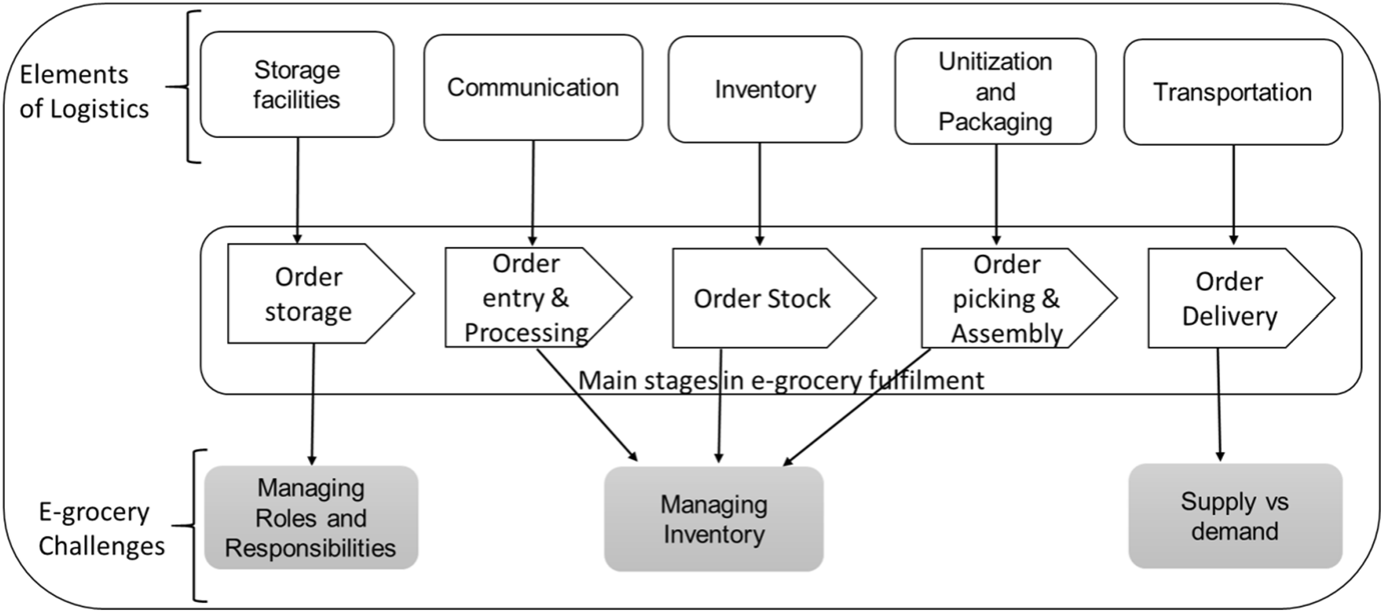
E-grocery stages fulfillment and e-grocery challenges [12]

Apart from the issues related to general e-business adoption, small and medium businesses in the e-grocery sector also have to contend with challenges related to managing logistics elements which constitute some of the most significant barriers to e-grocery adoption and affect businesses of all sizes [12, 33]. At worst, SMEs have limited resources to facilitate the key critical logistics elements of successful e-grocery retail activities, such as transportation, inventory, warehousing, picking, and packaging [15, 34]. Nevertheless, managing the logistics elements for grocery e-retail is more complicated than in any other retail sector [33, 35]. A common consensus surrounding the management of the retail logistics elements deduced from the practice of leading e-grocery retailers paints a complex undesirable picture of managing supply and demand, managing inventory, and managing roles and responsibilities, as shown in Fig. 1 [12].

Supply versus demand, and especially the transportation logistics element of e-grocery retailing, pose challenges related to the following: the suitability of online grocery options [36, 37], vehicle routing problems [38], attended home delivery issues caused by congestion and tight delivery windows [39], and cost-efficient consumer-oriented delivery issues [40]. The cost of e-grocery retail, such as the network complexity of the delivery process [41, 42] and thin profit margins [43], are also seen as barriers to entry.

Inventory management poses challenges related to the replenishment of online volume [33], the impact of the retail mix of the online store choice [44], the substitution of unavailable grocery items, handling, and sensory issues [45, 46], and freshness and quality of products [12]. The storage element requires different storage temperatures for grocery items [47] and poses legal trading issues associated with chilled and frozen products [16]. Furthermore, there are complex planning problems related to balancing marketing and operational considerations [48], replenishing online volume to stores, and keeping track of inventory [33].

Managing roles and responsibilities represents operational challenges in the e-grocery retail sector. According to Mkansi et al. [12], issues of prerequisite skills, insufficient human resources, human errors in the picking process, and retention have moved from the peripheral position of human resource management (HRM) to become formidable challenges for e-grocery managers. The issue of insufficient human resources is mainly felt when there is a sudden influx in demand that stifles supply capabilities. Put differently, the challenge of managing roles and responsibilities induces the problem observed by Fera et al. [49] of optimal order penetration against demand volatility. The picking process and order entry and processing pose challenges related to specialist skills, balancing costs with labor hours, limited operational capacity, technological challenges, and human errors [12]. In attempting to mitigate the challenges, large grocery e-retailers draw staff complements from other departments. This begs the question: how do small and medium e-grocery retailers contend with limited staff and fund management roles and responsibilities?

In a country with townships, such as South Africa, these challenges are heightened by the external factors and nature of the infrastructural issues surrounding SMEs in developing countries, such as telecommunication infrastructure, low income, low credit card penetration, and immature logistics infrastructure, which affect the timely supply of goods [25, 50–52].

Researchers who explored how e-grocers manage the challenges across the logistics elements focused on the practice of large businesses [12, 33, 44]. Unfortunately, the literature on the adoption challenges and coping strategies of specific mobile application e-grocery models is scanty and fragmented. Previous research has either revealed the logistics processes and different mobile application e-grocery models (e.g., mobile application retail, fast-moving consumer goods (FMCG)/brand, and wholesale models) [15] or has explored the acceptance of non-e-grocery mobile commerce in the distinct stages of adoption. From an e-business/e-grocery adoption stance, it is of utmost importance to understand how SMEs cope with the comprehensive list of e-business/e-grocery adoption challenges that emanate from the literature to enable them to explore those challenges that fall outside the purview of literature, uncover coping strategies, and finally situate them within the apparatus of TOE towards a high grade of e-business/e-grocery adoption theoretical framework.

The general e-business adoption limitations faced by SMEs and e-grocery entry barriers associated with high-cost barriers arouse curiosity into how small and medium e-grocery retailers manage the double-layered adoption challenges associated with the logistics elements necessary for successful e-grocery retail.

### TOE’s e-grocery adoption challenges

Although literature mainly presents e-grocery adoption challenges from the perspective of the logistics elements, most of the challenges can be consolidated and understood using the technology, organization, and environmental theoretical framework.

The technological construct of TOE describes internal and external technologies, such as hardware, software, internet, and payment processing tools, the possession of which is a significant determinant of e-business adoption. Beyond possession of the latter internal and external infrastructure, the technology needs to conform to Roger’s key attributes, such as perceived advantage, ease of use, compatibility, trialability, perceived importance of compliance, and perceived risks [53, 54]. The former technologies and latter attributes encapsulate the magnitude of the e-grocery challenges small and medium firms face in forging ahead to m-commerce. Therefore, a theoretical understanding of the challenges is essential.

From the evidence of the e-grocery literature, comprehensive challenges that relate to internal and external technological infrastructure for small and medium firms include, but are not limited to: network facilities, technical cost of ICT equipment [26, 27]), software for secure payment [29], internet [28], bespoke information warehouse, systems for traffic management or routing [38], systems for inventory management (i.e., to monitor the availability of stock, system to alert of possible substitutes, measure pick rates against targets), system to minimize errors [46], and maintenance and upgrades of the system [50].

TOE’s organizational context describes the scope, size, managerial structure, internal needs, proactive technical orientation, and financial readiness necessary for adoption [54, 55]. A review of the e-grocery challenges reveals that the organizational context of small and medium firms pertains to issues of managing inventory and managing roles and responsibilities such as staffing, knowledge, specialist skills, human errors in the picking process [12], dealing with legal issues [56], quality and freshness of e-groceries [44], logistics resources for timely supply and distribution of groceries [41, 42], the profitability of business models [43], and financial resources to address the challenges [28, 50].

TOE’s environmental construct is related to external factors such as government regulations, industry, and competitors [14, 55]. From the e-grocery challenges, environmental issues involve government support in terms of information [14, 55], legal frameworks, road network infrastructure, telecommunication infrastructure [25, 50], and dealing with competition.

**Fig. 2:**
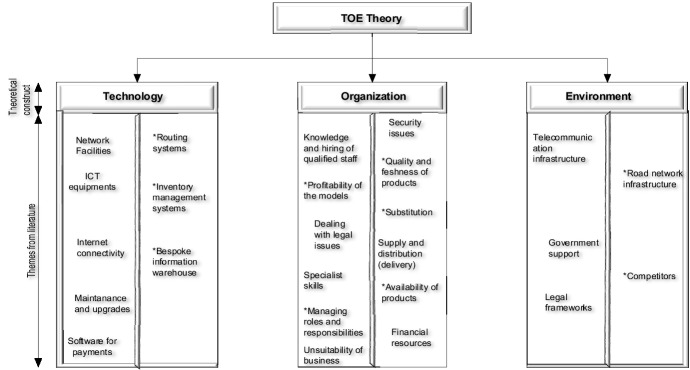
TOE’s e-business/e-grocery adoption challenges. Notes *means e-grocery adoption context challenges. *The technological* e-grocery challenges derived from literature are network facilities, ICT equipment, maintenance and upgrades, internet connectivity, routing systems, payment software, inventory management systems, and bespoke information warehouse. *The organizational* challenges that have been identified include knowledge, specialist skills, hiring of qualified staff, the profitability of the models, dealing with legal issues, security and trust issues, quality and freshness of products, availability of products, substitution, the management of roles and responsibilities, supply and distribution (delivery), financial resources, and the unsuitability of business. *TOE environmental* e-grocery challenges relate to telecommunication infrastructure, government support, competitors, road network infrastructure, and legal frameworks

The repositioning of e-business and e-grocery adoption challenges within the three TOE constructs is illustrated in Fig. 2. It captures the dynamic and contextual barriers faced by e-grocery SMEs, which, in turn, requires reformulating the adoption models based on the specific industry context. The intricacy and pervasiveness of e-grocery challenges have created a multifaceted barrier that is innately tricky for SMEs to overcome, especially those with extensions in townships and rural areas. This is mainly because the e-grocery operation is deemed an urban experience due to low order volumes and the high costs of assets required for the necessary distribution (stores, distribution centers, and transportation).

Although Mkansi et al. [12] provided evidence of e-grocery challenges and mitigating strategies from the top ten big global e-retailers, there is a lack of research that addresses the specific e-grocery challenges and mitigating strategies from the perspective of the SMEs to facilitate their development. Pollard and Morales’s [24] advice to scholars is to approach the SME environment and inform theory from the evidence generated from other small and medium firms’ practices. Following the latter advice, this study used qualitative research methods to shift the focus from leading e-grocery retailers to emerging small and medium e-grocery operators, especially those with extensions into townships and rural areas. The methodology followed in the study is discussed in the next section.

## Research methods

This research study is grounded in interpretative qualitative methods. In particular, we use the multiple case study approach to comprehensively describe the SMEs’ technological, organizational, and environmental challenges and the relevant mitigating strategies. The strength of the case study methodology in qualitative research and the exploration of the phenomenon in its context have been discussed at length by Yin [57] and Clark and Creswell [58].

### Case studies and sampling

The case sample consisted of eight of the possible 13 SME e-grocery retailers. All thirteen SMEs were contacted, but only nine responded. Of the nine, the CEO of one e-grocery withdrew, citing possible imitation by competitors, which left eight case study participants. For each sampled case, the participant was either a Chief Executive Officer (CEO) or a Chief Operating Officer (COO). The number of case studies and participants conforms to Eisenhardt’s [59] recommendations of four to ten case studies necessary to provide a rich understanding of the subject matter. The strength of purposive sampling in managing the extraneous variables necessary for internal validity was acknowledged by Daniel [60]. The sampled CEOs or COOs fully developed their respective distribution models, mainly were in charge of the strategies, and were involved in the activities related to the logistics elements of inventory, transportation, picking, communication, and in-transit storage.

From the eight case studies, five are micro-enterprises with only 0–9 employees, two are small enterprises with 10–50 employees, and one is a medium enterprise with 50–250 employees. Furthermore, four have operations in both urban and township areas, two solely in urban areas and two solely in township and rural areas. The key homogenous attributes of the case studies include the mobile-based grocery distribution model used and operations in the urban, township, or rural areas. Three types of mobile-based grocery distribution models were defined [see 15 for more details]. In the mobile application brand/FMCG configured models, the mobile e-grocers supply from FMCG/brand manufacturers, deliver bulk orders to a distribution center (DC) or the township and rural communities’ household garages for geographical penetration and temporary storage, break bulk the orders and deliver to the final or following destinations. In the mobile application retail configured models, the mobile e-grocers carry no stock and no stores, supply orders from big retailers’ stores, and deliver picked orders to the final destination. Lastly, in the mobile application wholesale configured models, the mobile e-grocers carry no stock and no stores, supply from a network of large wholesalers and big retailers’ stores, break bulk the orders, and redistribute to the final customers. Table 1 presents the characteristics of each selected company.

**Table 1:** Overview of selected companies

Mobile application e-grocery operator	Mobile application e-grocery distribution models, where	Sampling criteria								
	1 = Mobile application brand/FMCG configured models									
	2 = Mobile application retail configured models									
	3 = Mobile application wholesale configured models	CEO or COO	0–3 Years SME	3–5 Years SME	Township and rural operators	Urban operator	Innovator teams	O-9 Employees (micro)	10–50 Employees (small)	50–250 Employees (medium)
SME 1	1	*		*	*	*	*		*	
SME 2	2	*	*			*	*	*		
	3	*	*		*	*	*	*		
SME 3										
SME 4	2	*	*		*	*	*	*		
SME 5	2	*	*			*	*			*
SME 6	3	*	*		*		*	*		
SME 7	2	*	*		*		*	*		
SME 8	3	*	*		*	*	*		*	

### Data collection

Semi-structured interviews were the primary data-collection methods used to attain an in-depth understanding of the nature of the technology, organization, and environmental challenges experienced by small and medium mobile application e-grocery retailers and the associated mitigating strategies. We further engaged with the different e-grocery mobile applications to observe how they operate, and some of the CEOs showed us a demo of their application to illustrate how various features help them to cope with some of the challenges (e.g., quality and freshness of products, payment mode, and more).

The strength of semi-structured interviews in soliciting a deeper exploration of context has been acknowledged by various scholars [57, 61]. The e-grocery challenges situated within the TOE theoretical construct served as the framework for the interview guide. Prior to the interview, the guide and critical constructs were explained, including the methodology to ensure trustworthiness and transferability, as considered by previous scholars [57, 62]. All the interviews were recorded on computer audio, transcribed, and shared with participants to ensure credibility, completeness, validity, and clarification for convergence of the constructs discussed, in line with recommendations by Yin [57] and Rossman and Rallis [63].

Although the case study methodology is criticized for generalizability [57, 61], most coping strategies used to lower the e-business/e-grocery adoption barriers can be transferable to other contexts and potentially inspire other small firms towards e-business adoption.

### Data analysis

Following the confirmation and accuracy of the transcripts, they were uploaded for analysis into Atlas.ti, a computer-aided qualitative data analysis software that offers functionalities and flexibilities for different coding schemes.

**Fig. 3:**
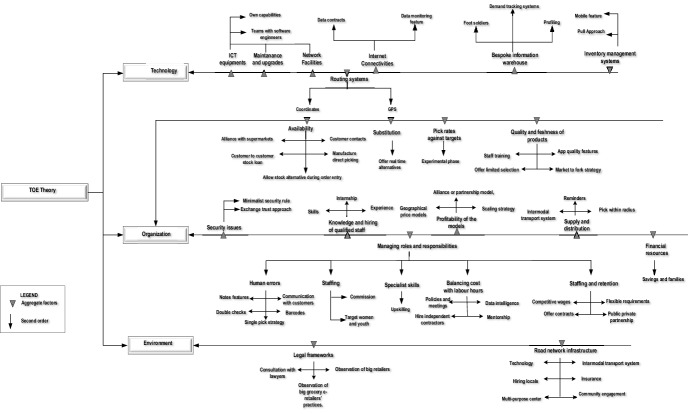
Data coding

Figure 3 illustrates the coding process that was followed. Previous scholars [64, 65] considered the coding process to be hierarchical axial coding. The first coding used was open coding, which opens up the transcripts to capture meanings and derive the codes used by the small firms to articulate and describe the double-layered challenges pertaining to their technological, organizational, and environmental context. Examples of open codes for enabling factors related to the knowledge of the organizational context are “…lead app inventor, I am the developer, my business partner heads up coding team”. Open coding is highly recommended by Babbie [64], who cautions that failure to open transcripts compromises the analysis and presentation of findings. The second coding represented the short phrases reflecting the salient descriptions offered by various small firms in a similar context (descriptive code). For example, open codes such as “…lead app inventor, I am the developer, my business partner heads up coding team” are summarized in the short phrase ‘skills.’

Descriptive codes were crucial for combining multiple open codes that can be further linked to the research instrument themes derived from literature, such as: ‘skills’ are linked to the aggregate code or thematic code ‘enabling factors related to the availability of ICT skills, and qualified personnel,’ derived from literature. Hence, the third coding represented aggregates or thematic codes that envelop and align descriptive codes to literature themes. Previous scholars regarded the coding process as hierarchical axial [64, 65]. The last or fourth coding process linked thematic or aggregate codes to the three theoretical constructs of the technology, organization, and environment context. For example, aggregate codes ‘skills are linked to a theoretical organizational context.’

The four coding processes that were used provide an analytical map representing a varied repertoire of the qualitative coding and analysis followed in this study are detailed further in Appendices 1, 2, and 3, included as supplementary materials.

## Findings

This section discusses the technology, organization, and environment e-grocery challenges and associated coping strategies.

### Technological e-grocery challenges

The findings related to the TOE’s e-business/e-grocery adoption challenges, as presented in Fig. 2 for technology, are addressed in this section. Table 2 summarizes the coping strategies used to overcome these adoption challenges.

**Table 2:** TOE's technological challenges and coping strategies

TOE construct	Challenges	Coping strategies	Mobile application models		
			Mobile app retail	Mobile app brand/FMCG	Mobile app warehouse
Technological	Network facilities, ICT equipment, and maintenance and upgrades	Interface with organizational construct strategies of specialist skills, and team diversity that consists of software engineers and developers	●	●	●
	Internet connectivity	Negotiating with media center to be a master distributer of data			●
		Use built-in features to monitor and optimize data usage	●	●	
		Accept and pay the cost	●		
	Routing systems	Exploit capabilities of Google Maps	●	●	●
		Foot agents captures coordinates during order process, which later feeds to delivery addresses		●	●
	Software for Payments	Use a combination of flexible payment methods such as: electronic wallets, electronic funds transfer (EFT), speed point, cash on delivery, bank deposit, credit, and PayPal	●	●	●
		Stokvel society model			●
	Inventory management systems	Develop built-in features that offer visibility of stocks		●	●
		Substitution features on the mobile app (interface with organizational context strategy for managing substitutions)	●		
	Bespoke information or data warehouse	Tracing and tracking of demand by location	●	●	●
		Understand stock consumption patterns	●	●	●
		Profiling of customers		●	●
		Link mobile application to a web-based platform which uses machine learning to understand stock consumption patterns that need to be dropped to different townships		●	

#### Network facilities, ICT equipment, and maintenance and upgrades

The findings reveal that fewer challenges are associated with the technological context of network facilities, ICT equipment, and the maintenance and upgrades necessary for e-business adoption across all eight e-grocery SMEs. This is mainly because all eight case studies comprise teams led by or have software engineers and developers behind the e-grocery mobile application-based distribution models. As such, development, maintenance, and upgrades are addressed in-house, and mobile phones and laptops for coding remain the critical ICT equipment, as highlighted by one of the eight CEOs:

“My business partner heads up the coding team that focuses mainly on smartphones because they have proved very resourceful in South Africa, but also across Africa. We know that every future smartphone will become more acceptable and used.” [CEO of the mobile application brand/FMCG model, SME #1].

#### Internet connectivity

The challenge of internet connectivity is of great concern for the e-grocery SMEs sampled in this study. The data costs, rather than the availability of infrastructure or service providers, are quite expensive in SA. A COO operating in urban and rural areas explained how they navigate data costs: “Data is a costly expense of our business, but we self-built our application, so we know exactly how much of data it uses, and we optimize the usage of data.” [COO of the mobile application retail model, SME #2].

In seeking to address the challenge, another CEO of the SME indicated that “we are negotiating with Media Centre to become a master distributor so that we can get data cheaper.” [CEO, mobile application wholesale model, SME #3].

In addition, two SME firms have built-in features that enable them to monitor and optimize their mobile applications’ data usage. The other five SMEs appear to be paying the costs because the internet is an indispensable part of their business models. This finding suggests that internet connectivity is an inhibitor for accelerating the growth of SMEs, especially those businesses where the internet is a dependent demand, as explained by the founder of the SME below.

“Data is a costly expense of our business, but we self-built our application, so we know exactly how much data it uses, and we optimize data usage. Also, we have features on the order entry agents’ phones that measure the data usage of our application. It is all built into one app, and so we monitor that one app for mobile usage data.” [CEO of the mobile application retail model, SME #4].

#### Routing systems

Another vital system factor of the technological e-grocery distribution challenge is the mobile application’s routing capabilities of coping with tight delivery windows, minimizing fuel, and avoiding traffic and congestion in highly built-up areas for attendant home deliveries. The issue of routing vehicles in e-fulfillment is accentuated by the trade-off between limited delivery vehicles and unstructured streets or/and house numbers in townships and rural areas against the pre-determined time slots promised to customers, primarily to locations far apart.

Most SMEs praised Google Maps’ capabilities to help them navigate to different locations. The brevity of most SMEs’ expressions was captured by one of the COOs and founder of SME #2, expressing that “we use the google map application to locate the address of where to deliver.” In attempting to deal with unstructured streets and house numbers, some SMEs intelligently use their foot agents to capture the coordinates of accurate addresses for deliveries during order entry. This is particularly apt in townships and rural areas where foot agents do order entry for their higher digitally-challenged market segment. “We optimize around the pin drops and plan the best route and number of deliveries in the same vehicle.” [Founder/CEO of the mobile application wholesale model, SME #3].

#### Software for payments

Software for payments is one of the notable challenges SMEs face, especially since it breeds trust and privacy issues. The case studies used eight flexible payment methods: electronic wallets, electronic funds transfer (EFT), speed point, stokvel society model, cash on delivery, bank deposit, credit, and PayPal. Small and medium e-grocery firms mostly use the methods above of payment, in combination or separately, to overcome not only barriers related to payment software but also trust, privacy, financial exclusion, and security issues. For example, three SMEs of mobile application retail and wholesale configured models use a combination of e-wallets and cash on delivery expressed similarly as: “We have two payment methods, they can deposit it into any of our accounts such as an e-wallet system that is transferred into us. Otherwise we do cash on delivery”. [CEO of the mobile application retail, SME #5].

However, some participants experienced accounting issues related to cash in transit. In particular, the loss of receipts affects the audit trail and balancing of financial statements. As such, a petty cash card was used to mitigate such accounting problems.

The majority of the participants were not in favor of the cash payment option for safety reasons. Instead, the majority embraces more payments by e-wallet, EFT, and credit card. Delivery is only made once cash is cleared and reflected by the bank.

The payment options reveal how the flexible options made possible by retail banks enable SMEs to leverage mobile commerce to generate new markets and employment opportunities. Thus, the retail banking options available to their varied socially disadvantaged market segments indirectly create a fertile environment for SMEs’ mobile commerce and digitally challenged markets at an unimaginable scale.

#### Inventory management systems

The study sought to understand the e-grocery mobile applications’ ability to manage stock, considering that they heavily depend on major retailers, wholesalers, and manufacturers' stock. Most small and medium e-grocery CEOs that use mobile application retail configured models reported, “Right now, we do not have issues with out-of-stock because we have always been substituting.” However, two firms using the mobile application wholesale and mobile application FMCG models, respectively, similarly offered insights into their plans for developing features that will link them with a buying group. The featured link will give small and medium grocery retailers visibility of stock levels as the orders are placed in their app.

“We once had talks with a buying group, and they were willing for us to create a link from our app to their distribution center so that if we place an order, we should know what is available there. The feature will be a back-end for us to know the buying group's stock levels and not for anybody else to download”. [CEO of mobile application wholesale model, SME #6].

#### Bespoke information or data warehouse

The findings related to bespoke information revealed varied responses based on the specific e-grocery mobile application models. SMEs that use the mobile application brand/FMCG models provide insight into their e-grocery mobile applications’ capabilities to offer data related to product forecasting, market segment information, and tracing/tracking of demand by location. Regarding stock forecasting information, one small firm has linked its e-grocery mobile application to a web-based platform that uses machine learning to understand stock consumption patterns that need to be dropped to different townships.

“Our system has a web-based platform, and it uses machine learning in terms of stock ordering. So, we have started to understand exactly how much or how little we need to order. It helps our model. We can turn it around very quickly, enabling a positive cash flow situation. For our brand manufacturer partners, we supply reports like rates of sales and all kinds of data flow, which is extraordinarily valuable in a market. Soon as we have price increases, we can see exactly how it affects the market”. [CEO of the mobile application brand model, SME #1].

In terms of tracking demand, six e-grocery mobile applications across all three e-grocery mobile application models commonly indicated that they can track and study demand by location. Furthermore, the location feature and map integrated into the e-grocery mobile application validate whether the address is legitimate.

*“*One thing we are tracking in our App is the demand by location. So, when a customer sign-up, the first thing to select is the location. This ensures that people in Cape Town do not expect us to deliver when we are only based in Johannesburg. But far more than delivery by location, we can store a lot of interesting product data, so we can see where all our demand is”. [CEO of the mobile wholesale configured model, SME #8].

Some SMEs use a non-system approach, such as profiling, to supplement the tracking of demand by location and to study their target market in townships and rural areas. Profiling of customers is embedded in their order collection and entry process information about location and family dynamics necessary to predict the family's different needs. The findings suggest that beyond bridging the digital divide and creating inclusive e-grocery mobile application commerce models and opportunities, SMEs are brewing pots for digital cognitive skills, such as sense-making, curiosity, and probing, which are crucial for mobile commerce.

“We take young people to a target area, and we do it every week, once or twice a week, and they go door to door, meeting customers and telling them about us. They are soldiers for marketing, and every Tuesday, they are required to come with a customer profile to our meeting. For example, whom they meet, where they live, do they have dependents, are they married, what level of education they do have, do they have an income, what is their job, how much they earn, what kind of products do they buy, how old are they, are they religious profile”. [CEO of the mobile application wholesale model, SME #3].

### TOE’s organizational e-grocery challenges

This section discusses the findings related to the e-grocery challenges that were experienced, as presented in Fig. 2, for the organizational aspect. Table 3 summarizes the coping strategies used to overcome these adoption challenges.

**Table 3:** TOE's organizational challenges and coping strategies

TOE construct	Challenges	Coping strategies	Mobile application models		
			Mobile app retail	Mobile app brand/FMCG	Mobile app warehouse
Organizational	Knowledge and hiring of qualified staff	Own internal skills as developers	●	●	●
		Orchestration of innovative teams with complementary skills	●	●	●
		Leverage sustainable labor approaches (e.g., subsidized learnership or internships)			●
		Commission strategy		●	●
	Security issues	Use of cash on delivery options with or without target limits and e-wallets	●	●	●
		Carry minimal amount of stock and/or cash	●	●	●
		Use of a pull model	●	●	●
	Quality and freshness of products	Shoppers’ training programs to place quality at the center of their picking activities	●	●	●
		Embedding quality specification features on mobile applications that provide customers with freshness specification options at the point of order	●		●
		Use of farm-to-fork approach	●	●	
	Availability of products	Seek customers’ alternative options through direct calls	●		●
		Search for missing products anywhere for the promised price and delivery delay	●		
		Customer-to-customer loan in exchange for a credit note			●
		Seeking availability from local supermarkets through direct calls	●		
		Built-in features in the mobile applications to issue alternatives for product with high risk of unavailability	●		●
	Substitution	Real-time suggestion to customers in case of products unavailability	●		
		Use a proactive method where customer recommend alternatives for those products that have high probability of unavailability			●
	Managing roles and responsibilities: human errors	Built-in features that enable adding notes to the order			●
		Use of bar codes to confirm order accuracy	●		
		Fostering single-picking over multiple picking	●		
		Bear the brunt of re-delivery and the associated costs	●		
	Special skills	Offer competitive rates	●	●	●
		Do not contend with PDB license	●	●	●
	Financial resources	Loans from family and friends	●	●	●
		Savings	●	●	●
	Profitability models	Geo-pricing strategy			●
		Uber partnership	●		
		Mini-distribution centers (DCs) (Spaza shops partnership) Warehousing		●	●
		Shared value student model	●		

#### Knowledge and hiring of qualified staff

Contrary to the view that small and medium enterprises usually lack knowledge and struggle with enabling factors such as specialist skills and the hiring of qualified staff, this study found a wave of knowledgeable mobile application innovators who understand how to maximize their skills and demonstrate the ability to seek partners with complementary skills. All eight small and medium grocery firms were composed of teams that included developers and software engineers. One of the CEOs highlighted:

“There are three of us on the team, my colleague who is the lead in inventing the App, myself (CEO), and also cover a bit of those App development, website, and the stuff that the outside world does not see many times, which is a lot of our tools like control orders in the system.” [CEO of the mobile application retail model, SME #7].

Notably, three of the SMEs’ CEOs appear to harness experience gathered from their previous employment with various retailers and FMCG companies. The findings highlight some essential traits of e-business and e-grocery adoption that can better explain e-grocery start-ups’ innovativeness and digital entrepreneurs’ idiosyncratic traits, such as pre-entry or high levels of industry-specific experience.

“I am the developer and have used business analytics. I am the COO in charge of operations. Before this business, I worked as an engineer, logistics, and manager in consulting at Boston Consulting Group. While there, I worked with some of the big retailers in South Africa; I cannot mention their names because of some policies from them. I have seen their operation models and how they plan on dealing with an online business.” [COO of the mobile application retail model, SME #2].

Apart from their skills as developers and the orchestration of innovative teams with complementary skills, the two SMEs leverage sustainable labor approaches such as subsidized learnerships or internships. While the internships offer a cost-effective hiring model for the SMEs, it further highlights the importance of government agencies, such as the SETAs, in fostering SMEs’ e-business adoption, public and private partnership, and in addressing social problems, such as unemployment, which simultaneously improve the well-being of socially disadvantaged groups.

“We use learnership, and the cost of our labor is subsidized. SETA has discretion grants, so if your training and upskill people, they pay them a stipend, and they pay you (the business) for the training”. [CEO of the mobile application wholesale model, SME #3].

Some small and medium e-grocery retailers manage the issue of staffing through a commission strategy, which is crucial for generating rebates with manufacturers. However, some SMEs use commission as an add-on to a basic salary, but they specifically target women, independent contractors, and youth. The more business-to-business (B2B) customers are signed up, the higher the credit and the commission. “Our people are commission-based and write their checks. They get the sales and try and push as much as possible and help us generate good rebates with manufacturers…” [CEO of the mobile application brand model, SME #1].

#### Security issues

The literature has discussed in depth how the security and trust issues related to the e-commerce system are barriers to e-business adoption. However, for SMEs in the e-grocery sector, security and trust issues extend beyond the mobile application (m-commerce) to include cash security in transit and products. In terms of mobile commerce, the challenge relates to customers’ trust in processing their credit card information in the mobile application. In response to the trust issues, six SMEs have introduced cash-on-delivery options and e-wallets. However, the cash-on-delivery option breeds its own security challenges, considering the high crime alert in the townships of SA. As such, two SMEs introduced a daily cash transit target amount, which a driver can carry, and anything more than that amount must be deposited through an ATM. In terms of the security of grocery stock in transit, some small grocery retailers prefer to carry minimal stock rather than large volumes, even if it means several costly delivery journeys.

The findings highlight some trade-offs of adopting the e-grocery model in townships with complex security issues. Fortunately, the case studies use a pull model. Therefore, there is a low risk of dead stock. Also, they never have to worry about lost income because their e-fulfillment is from any big grocery retailer, which gives them freedom of choice and quantities: “We keep everything small, we do not carry huge amounts of stock or huge amounts of cash. We would rather do multiple small deliveries than one big delivery of large amounts.” [CEO of the mobile application retail model, SME #4].

#### Quality and freshness of products

With respect to managing the quality and freshness of products, small e-grocery retailers operating a mobile application wholesale configured model reported an experience with one of their partners who sold them outdated products under the disguise of assisting with picking and packing. Since the discovery, the e-grocers have managed their own operations. The experience reveals some conflicting objectives of trust and dependency between trading partners.

“When we started, people would sell us short-dated stock. I call it being baptized with fire and water, and we earned our scars. We get our stock directly from a wholesaler, and they pack for us in advance, but when you think that this person is trusted and then they sell expired stock or those that are left with few days to expire, and only to go to a wholesaler and find very fresh stock is painful. So, we had to learn quickly, and we discovered that this a very shrewd industry”. [CEO of the mobile application wholesale model, SME #6].

The key mitigating strategies for dealing with freshness issues are training shoppers, embedding quality specification features on mobile applications, and the farm-to-fork approach. Some small e-grocery retailers appear to have gathered research intelligence relating to customers’ reluctance to adopt e-grocery shopping. Hence, they have initiated training programs for shoppers to place quality at the center of their picking activities. This offers an interesting insight into the emphasis of their objective, which affects large grocery retailers differently. Unlike large grocery retailers, whose objective is to shift stock with a minimum shelf life to avoid waste, SMEs do not own stock. Therefore they can afford to always prioritize quality to ensure the best experience for their customers.

“Quality is one of the highest priorities, and we always ensure we keep within dates. We are incentivized differently from traditional grocery retailers. For example, Pick n Pay online and Woolies online are incentivized to finish their stock, which is why they will give you the last tomatoes, but we do not own stock, hence, the priority of quality. Our incentive is to give the best customer experience, and we do not have the pressure of having to finish the stock. So, our guys pick the best produce and have been trained with that in mind”. [CEO of the mobile application retail model, SME #7].

Some SMEs have embedded quality features that allow the customer to specify the ripeness and freshness of their desired products at the point of order. The strategy helps in meeting the expectation of the e-grocery customers. The insights reveal how technology serves some hard-to-achieve essential touch and feel senses. Understanding the effectiveness of this strategy can help achieve a balance between cost and service, which is necessary for retaining customers and encouraging more people to adopt e-grocery shopping.

“Our app enables our customers to comment on the quality they prefer when they place the order in the basket. That way; we can guarantee the good quality of the product we are selling”. [CEO of the mobile application wholesale model, SME #8].

Also, a farm-to-fork strategy is used by three SMEs to mitigate the issue of the freshness of products. Beyond freshness, the strategy is one of the few that empowers and supports local markets, is necessary for keeping jobs, and contributes to the country's gross domestic product. Especially, in a country like South Africa, where small farmers are excluded from the large retail chains and usually have to contend with limited infrastructural resources.

“Our company does not keep inventory, so we buy on demand. When you go to our app and our website, you will see that we promise to deliver before twelve. We buy from the market in the morning and deliver the same day before twelve. We supply straight from the market, which is how we manage freshness”. [CEO of the mobile application wholesale model, SME #6].

#### Availability of products

The availability of products is one of the e-grocery challenges. Hence, the study probed small businesses for their mitigating strategies. The availability issue is far worse when dual fulfillment activities exist (in-store and internet picking). One small firm reported that 50–60% of e-grocery orders have one product out of stock. This is primarily fresh produce such as fruits and vegetables. At times, the issue of availability is a matter of difference in unit of mass (100 packs instead of 200 packs) requested by customers.

“I would say at least 50 to 60% of orders had at least one item out of stock. […]. So, sometimes it is very arbitrary. I think there are very common items that were out of stock. This is a key issue in grocery”. [CEO of the mobile application retail model, SME #4].

From the three e-grocery mobile application models, the CEO of the mobile application brand/FMCG configured model reports that there are no concerns regarding availability because of the advantage of sourcing directly from manufacturers “We buy directly from the brands' manufacturers and do not go into a wholesaler. We are at the start of the rich value chain, so we never have the issue of availability at the manufacturing level”. However, the mobile application retail and wholesale configured models have to contend with availability challenges.

Telephone contact is one of the strategies used to manage availability. This allows the manager to liaise with the affected customers to find alternative substitutions, and the feedback is communicated to the shopper to adjust accordingly. However, it is noted that most customers do not favor receiving calls during office hours but mostly respond to out-of-stock calls.

“We speak directly to the customers, although many people do not like being contacted during office hours, everyone responds to out-of-stock […] They are happy to give alternatives and appreciate that we informed them immediately”. [CEO of the mobile application retail model, SME #7].

Some e-grocers prefer to search for products anywhere for the price promised to customers. As such, the manager in charge gives customers 48 h to find the product and price desired by the customer. “Technically, everything is available, and we just have to find it. The challenge is where and how much time to find it at a good price. We ask customers to give us 48 h to source at a lower price for them”. [COO of the mobile application retail model, SME #2].

Another interesting perspective on managing unavailability is that of customer-to-customer stock loans. One small firm says that their B2B customers, such as Kota traders and bakers, can liaise with one of the customers with enough stock to loan them the products in exchange for a credit note, and the products are then later replaced. This approach is more practical in a township where client relationships are easier. The trend revealed an interesting aspect of trust and relationship building that is crucial for re-distribution trading patterns and displayed both between the e-grocery and the customer and the B2B customers.

“Sometimes, we have taken stock from one customer to give to another customer. Then take a new order from the customer to replace it. […] I do not know if it will be sustainable going forward. However, if you think about inventory as already distributed and all you are doing is move it to the point of need, then absolutely it is something that we should do easily in exchange of the credit note that says we owe you. […] It is all about the relationship, and we are lucky to get repeat customers, and we build a relationship with them. This kind of relationship allows us to do that, but as we grow the business, we must ensure that our agency continues to do those kinds of relationships because if they do not, some will be like do not touch my stuff. It is part of being in a township”. [CEO of the mobile application wholesale model, SME #3].

Another relationship-driven strategy was identified between the e-grocer and the managers of the stores, where they frequently pick their stock. In this case, the e-grocers call the stores' managers to see which supermarkets have stock availability. The emphasis is that their customers must receive accurate stock as ordered. The strategy is interesting because it is a clear example of coopetition between large grocery and small e-grocery retailers, highlighting another win–win situation.

“It involves a lot of work, we have meeting with the four managers of the local supermarkets. Through that relationship, we can check if the products are available and decide which supermarket to buy from. We do not change the products that the customer ordered. It is a matter of us getting the goods from one supermarket and the same location instead of running around or buying small portions and delivering them here and there. That falls into the promise to our customer that we get them the product they want”. [CEO of the mobile application retail model, SME #4].

An exception to all the non-digital strategies is the digital predictive method of one small firm that allows customers to choose alternative products while placing the order. The mobile application uses intelligence to issue alternatives for products with a high risk of unavailability based on historical data. The strategy is an example of the time and cost-saving strategies of the e-grocery retailer.

“We allow customers to give alternatives when they place an order on the app. If a customer picks an apple and we know this is frequently out of stock, we suggest alternatives, and s/he has three options or four options to choose from, based on our data. This is one way of predicting items that are frequently out of stock”. [CEO of the mobile application retail model, SME #5].

#### Substitution

Substitution is one of the e-grocery challenges intertwined with the unavailability of stock, but it is worse because it has cost implications*. *Unlike the practice of big global e-grocery retailers, not all small businesses can absorb the cost of substitution. Most of the e-grocery SMEs commonly highlighted what is captured by one CEO “We tell customers that we ran out of your brand, but alternatively, we can give you this one, and they decide whether they pay for it or not. We do not absorb any difference in costs”. [CEO of the mobile application retail model, SME #7].

The various mitigating strategies include real-time customer suggestions, pre-consideration features, and telephone communications. The real-time suggestion happens as shoppers pick products in the stores, and where there is unavailability, they send a message to the customer with alternative pictures of in-stock products. In instances where there is a price difference, some SMEs absorb the costs but intend to explore the ‘reserve amount’ strategy used by other international grocery retailers.

“When our shoppers do not find items ordered, they send customer information via the app, and it suggests alternative products by showing them the picture. The customer is given the option to accept or reject the alternative. We do not get something cheap, and our shoppers are trained to offer similar alternatives”. [CEO of the mobile application wholesale model, SME #6].

However, others use a proactive method by offering the customer the opportunity to recommend alternatives for products with a high probability of unavailability. “We know items that are more likely to run out of stock from previous order data stored in our system, so as they order, the system will ask what we must do if we do not find them. So, we can pre-consider what items to substitute with”. [COO of the mobile application wholesale model, SME #2].

#### Managing roles and responsibilities

The management of roles and responsibilities revealed challenges relating to human errors at the order entry, order pick, and order delivery phases of the e-grocery operation. From order entry, the limited brand knowledge of some of the foot soldiers taking orders on behalf of the digitally challenged market is of concern. As such, e-grocers constantly deal with incomplete information where a product is specified but not the unit mass or brand of the product requested. The implications are a waste of time and additional costs incurred for repeat order fulfillment.

“We have human error challenges in everything. We encounter errors during order processing on the app on behalf of the customer and order picking. Our young people from townships are part of a very uninformed market and uneducated about products, brand names, and pack size”. [CEO of the mobile application wholesale model, SME #3].

The small e-grocery retailers have added a feature that enables the foot soldier to add notes to the order, which helps mitigate against the issue of brands and mass. Looked at differently, the human errors encountered in the township and rural areas are a critical aspect of the co-creation of the e-grocery mobile application re-design because it serves as inputs that refine the mobile applications to suit the needs of their staff with limited knowledge, and that of their digitally challenged market. At best, it serves as evidence of the unplanned consequences of the co-creation and inclusion of voices that would not normally be considered in the design phase of technology.

“To address the situation where the order comes in, and it says my customer wants cornflakes, and I cannot find it on the system, we created a feature that allows our agents to add notes to their order. That way, we turn to know there is something a customer wants and go through the notes and say you want cornflakes? Which one? Which brand?” [CEO of the mobile application wholesale model, SME #3].

In terms of order pick, human errors were also experienced, especially when a shopper was addressing several customers’ orders at once. Issues of mixed orders and forgetting specified requirements of orders were most prevalent. Two strategies are used to mitigate human errors during order picking. The first strategy is using bar codes which confirm the accuracy of the other. “There were those challenges of picking up wrong items, but we addressed it by scanning everything. So, whenever shoppers pick the items, they scan the barcode to confirm that we have picked the right item”. [CEO of the mobile application retail model, SME #7].

Some e-grocers mitigate against picking order mistakes by fostering single-picking over multiple picking (one shopper picks for one customer at a time) and double-checks during picking and dispatch to customers.

“We try to make the process efficient by managing one customer’s order at a time. Shoppers only deal with one order at a time. They pick those items, pay for them, then start shopping for another customer, return to the store, and follow that process”. [CEO of the mobile application retail model, SME #5].

Human errors during order delivery relate to delivering smaller quantities than the ordered quantity. Small e-grocery retailers usually bear the brunt of re-delivery and the associated costs. “When the customer says this is not what I wanted, and it is small quantities, we apologies and re-deliver. It is part of our customer model”. Furthermore, there are reports related to products being dropped at the wrong address due to drivers’ assumptions and knowledge of frequent customers, which sometimes causes the driver to ignore the global positioning system (GPS) guide. In that case, the driver returns to the customer, re-collects, and re-delivers to the correct customers.

“The other day, the driver dropped the goods at the wrong customer. I was like, I do not understand, the GPS tells you the customers are two streets from each other, and I think the driver has been there before, so once he saw it, he just assumed he was returning there again. And that is a problem of the township sometimes when the houses are close to each other. They had to return, take the pack, and give it to the right customer. It was flour, and they both use flour”. [CEO of the mobile application wholesale model, SME #6].

#### Specialist skills

An additional challenge in e-grocery operation is the reliance on a few specialist skills, especially IT, drivers, and shoppers who understand the demand and peculiarities of e-grocery retailing. Fortunately, a critical IT dimension of e-grocery operations rests on the CEO. As for drivers and shoppers, some e-grocers commonly house up-skilling programs and, in most cases, work with the Harambee agency that offers focused training for blue-collar jobs. The upskilling program enables shoppers to become managers of personal shoppers based on the efficiency and speed of previous orders.

To retain their drivers for a sustained period some small e-grocery drivers across different models do not contend with the specific compliance license issues, hence, their ability to retain drivers for a sustained period. The CEO of SME #5, who operates a mobile application retail configured model, highlighted that “For us, they do not need a specific license, hence with Uber drivers need to have PDB licenses. PDB is a passenger caring license that you need as a taxi driver, which is not a requirement for us”. Further, small e-grocery retailers offer a competitive rate compared to some e-distributers. The latter highlights some of the challenges blue-collar workers face regarding decent salaries.

“The big issue in South Africa with drivers is their compensation. When we tried to speak to the people that work for like Mr. D, Takealot, and Uber, we figured out that they get paid R15 per delivery, which is on a first-come, first served. And they also do not get paid based on distance, so they arrive at the Takealot depot first thing in the morning. They are given many orders, and you do not make money for that day if you get there late. This is a big problem, and we want to compensate our people better. So, our delivery guy pecks are slightly higher, where Mr. D pays R15, we pay R20… “ The problem in this industry is that businesses focus on maximizing staff as much as possible for less pay. And then you end up with a situation of very unhappy drivers. We believe that happy drivers equal happy customers”. [CEO of the mobile application retail model, SME #4].

#### Financial resources

Trade finance is one of the most significant challenges small e-grocery retailers face. In fact, none of the eight case studies interviewed has benefited from government or retail bank funding. Most used their savings or loans from family and friends to start their e-grocery businesses. The acute concern is the lack of guarantee, besides cash flow problems. As such, families and personal savings appear to be critical engines for bridging the TOE e-grocery’s financial adoption challenge. Nevertheless, SMEs appear to be swimming against the tide and making headway into a complex digital and resource-demanding market.

“Financial support from private companies, investors, or government institutions is terrible. We started this business with the little money we had, and we borrowed from families sometimes. It affects us and our plans to expand”. [COO of the mobile application retail model, SME #2].

#### Profitability of the models

The study derived five models of ensuring the profitability of e-business/e-grocery mobile application fulfillment models against the high transportation cost and thin profit margins: geo-pricing strategy, Uber partnership, mini-distribution centers (DCs), warehousing, and shared value student model. Most profitable strategies relate to specific e-grocery mobile application distribution models. For example, the CEOs that used the mobile application wholesale configured model decided on the geo-pricing strategy upon realizing that the prices of groceries were relatively high in Tshwane (Pretoria) compared to Johannesburg. Their markup calculation is based on the cost of the products at the warehouse nearest to the customers’ location.

“The price of products is higher in Tshwane to source, compared to Johannesburg. But not everything, so we are now pricing based on the area where we source. We reached a point where the customers’ price of products depends on their location”. [CEO of the mobile application wholesale model, SME #3].

A CEO using the e-grocery mobile application wholesale configured model forged a relationship with a B2B spaza shop customer with a high volume of weekly orders, who, in turn, acts as a mini distribution center. The strategy enables the e-grocer to ensure a constant supply to the market without having to incur daily transportation costs for low order volumes. In that context, customers place their orders on the mobile application, but the groceries are picked, packed, and delivered from the spaza shop by the spaza owner to the customers within an easy radius from the shop. Again, the strategy is considered a win–win for both, as the e-grocer saves on order fulfillment costs, and the spaza shop owner receives credit and discounts on grocery stock to balance the running costs of the last mile. In some locations with either high or low density, the e-grocery mobile application wholesale configured model also adopted a similar model to that of mini-DC but in terms of wholesalers. The wholesaler can list products on their mobile applications, which they deliver to customers, and the e-grocer and the wholesaler share the profits.

“We looked for big spaza shops and got one guy that buys R32 000 worth of stock every week. We asked the spaza to help supply people around his area and put them in a business where they become like a mini-DC for us. They help us save costs, instead of going daily to the same person that orders low volumes, they can get the stock from a micro-DC. The spaza transfers everything electronically, we give him a seven-day credit. As a reward for his partnership, we give him a discount that also makes sense in absorbing some of his running costs”. [CEO of the mobile application wholesale model, SME #8].

One of the CEOs using the e-grocery mobile application retail configured model explored a partnership with Uber that will address the delivery aspects related to groceries across SA. The approach was considered a win–win for both the e-grocers, who can scale without transport constraints and Uber for constant deliveries. “We have been shortlisted to pitch to Uber to help us with deliveries to households, enabling us to expand the system and business to other big cities like Cape Town and Durban.” The CEO of a retail-configured model with university students as the target market also offered a different perspective on a partnership. The CEO conveyed plans for a partnership with landlords of the students’ residential areas where they can advertise and create micro jobs for students. This is another demonstration of SMEs’ role in creating jobs.

### Environmental e-grocery challenges and mitigating strategies

From the literature, the TOE environmental e-business and e-grocery challenges relate to telecommunication infrastructure, government support, competitors, road network infrastructure and legal frameworks. The study found that of the five environmental challenges presented in Fig. 2, the road network infrastructure, which relates to supply and delivery challenges that also interface with organizational context challenges, is most prevalent. Legal issues are challenges that are addressed from the TOE organizational context, but they also emanate and interface with the TOE environmental context. Table 4 summarizes the coping strategies used to overcome these adoption challenges.

**Table 4:** TOE's environmental challenges and coping strategies

TOE construct	Challenges	Coping strategies	Mobile application models		
			Mobile App retail	Mobile app brand/FMCG	Mobile app warehouse
Environment	Road network infrastructure	Use of an intermodal transport system involving vans and other means (e.g., bicycles, wheelbarrows, etc.)			●
		Protect against riots by purchasing insurance	●		
		Working with local drivers accustomed to township driving patterns	●	●	●
		Exploring future technologies such as drones, solar powered multi-purpose center and community strategic alliances			●
		Exploring e-cycling program and partnership with the Council for Scientific and Industrial Research (CSIR)			●
		Pick from the nearest mall or shopping center	●		
	Dealing with legal issues	Consultation with lawyers	●		●
		Observation of big grocery e-retailers’ practices	●		●
		Awareness of policies and current affairs	●	●	●
		Use of frozen bags with prescribed temperatures for frozen products	●		
		Offer credit in exchange for another purchase			●
		Tries to align with labor act to some extent			●

#### Road network infrastructure

The SMEs' description of infrastructural challenges centered more on the road network, congestion, riots and breakdown, and van asset challenges that interface with organizational context challenges. In terms of road network infrastructure, the spatial inequalities of rural areas, which are different from urban areas, are problematic when aiming to optimize e-grocery deliveries. The challenge with some rural areas, like those in the Eastern Cape and Kwa-Zulu Natal, is that houses are spatially dispersed, which not only excludes rural people from economic opportunities but also makes it hard for small e-grocery retailers to optimize supply in terms of time, fuel, and target deliveries. At worst, there is no clear road to reach the customers in some areas.

“The infrastructure is not a problem in urban areas as it is in rural areas. The biggest problem with infrastructure in rural areas is that it is geographically dispersed, like the rural Eastern Cape and KZN. We have villages with one customer here and another over there, but there is no road. The people use wheelbarrows to carry goods. So, that is part of the problem, and now we are looking at different transport modes such as bicycles, but if you cannot get a bicycle into that area, what else can we do to be able to move things around? But for now, if it means we, the supplier, must physically wheelbarrow the products, we will ensure we get the product to the last point". [CEO of the mobile application wholesale model, SME #3].

In mitigating road infrastructure challenges, some small e-grocery retailers use an intermodal transport system, such as bulk deliveries by van to the center of the specific rural area(s), where bulk orders are separated and re-loaded into bicycles and wheelbarrows. Therefore, some e-grocers are looking into the future for technologies such as drones, solar-powered multi-purpose centers, and community strategic alliances. Regarding technology, one small firm said, “Today, we had a conversation with one of the guys who is from the world of navigation, drones, and geolocation. We discussed what kind of drones or technology we need to address road infrastructure in rural areas.” [CEO of the mobile application wholesale model, SME #6].

Besides road infrastructure challenges, other SMEs report on congestion, riots, and breakdown of vans. The congestion issue is more prevalent in townships, where minibus taxis appear to use unconventional driving methods that disrupt the on-time delivery of e-groceries. One small firm's sentiments are, "We experience congestion in townships because taxis drive their way and stop in the middle of the road." [CEO of the mobile application retail model, SME #5].

The issue is exacerbated when there are riots or the sudden breakdown of the van. The issue with riots is that protesters are more likely to vandalize the delivery van, which significantly reverses the progress of the small e-grocery retailer, especially considering how long it takes for a small firm to be able to afford a van.

"Transportation is always a risk factor because if it breaks down and needs to be serviced, people can throw a stone at your car, mostly when there are riots. But we have insurance for it, even though sometimes it feels like it is money being thrown down the drain." [CEO of the mobile application retail model, SME #7].

Subsequently, some SMEs mitigate the congestion issue by working with local drivers accustomed to township driving patterns. The verbatim expression of the small firm #4 operating a mobile application retail model is that "We hire people in that particular township, so they know how to navigate around the issues." Another small e-grocery tried to invest in a van, however, as the demand increased, scaling deliveries with one van asset was a challenge.

#### Dealing with legal issues

The current and prospective legal issues that small mobile application e-grocery retailers revealed are compliance in terms of alcohol, returns, tobacco, food quality, credit offerings, human resource issues related to micro jobbing, and trade unions. The issues related to liquor and tobacco are that although there are laws that prescribe the condition for selling and distributing liquor and tobacco products, the lines are blurred when it comes to the e-grocery business. This applies to a small e-grocer whose supply and distribution are independent from big grocery retailers where stock is sourced, and the license is held.

The mitigation strategy is consultation with lawyers, observation of big e-grocery retailers' apps, and awareness of policies and current affairs published by the liquor board. This means that e-grocery mobile applications are part of the digital revolution that alters how alcohol is sold and distributed to customers. Ultimately, this calls for a re-interpretation of the law for digital markets.

The challenge for those experimenting with credit as a payment method was the National Credit Act. They needed to determine the implications of delivering food and collecting payment later. However, the matter did not warrant much action since the payment model was not sustained. The participant's verbatim expression is, "The National Credit Act cannot be avoided because if I give a customer food and he or she pays me later, it means I am giving them credit. We must figure out how it works to ensure we are not left liable for breaking the law". [CEO of the mobile application wholesale model, SME #3].

Another challenge is posed by returns, especially in the case of unattended home deliveries, where the customer may be unavailable to sign for grocery products. Returns generate costs; at worst, the e-grocery customers purchase is linked to the small e-grocery retailer, not the big grocery retailer where the grocery was sourced. As such, small grocery retailers' liquid business model with no storage place and stock make it quite challenging to deal with returns, which the Consumer Protection Act mandates. As such, small e-grocery retailers offer credit in exchange for another purchase on the platform. While there are infrequent returns, it is at the center of the e-business/e-grocery adoption challenge.

### Other emerging e-business/e-grocery challenges

This section discusses other emerging field-based challenges faced by mobile application e-grocery retailers that are, to the best of our knowledge, unexplored in the literature. These emerging mobile application e-grocery adoption challenges focus more on the organizational construct of TOE than technological and environmental contexts. Table 5 summarizes the coping strategies used to overcome these adoption challenges. The township and rural markets present two sides of the same coin for small and medium e-grocery retailers. On the one side, it presents an untapped market waiting to be served, and on the other side, a cost implication since the digitally challenged market requires extra personnel to process orders on their behalf*. *"I think the teething issues that we used to have been customers' understanding of how to use an application, but driving a ground force team to conduct sign-up helps." [CEO of the mobile application wholesale model, SME #6].

**Table 5:** TOEs’ emerging organizational challenges and coping strategies for e-grocery SMEs

TOE construct	Challenges	Coping strategies	Mobile application models		
			Mobile app retail	Mobile app brand/FMCG	Mobile app warehouse
Emerging Organizational	Digitally challenged market (unable to place orders on the app)	Deploy foot agents		●	●
	Reluctance in terms of e-grocery adoption (trust issues)	Raise awareness of the potential and convenience of e-grocery shopping			●
	Customers’ behavior toward electronic market, i.e. unavailability of customers during delivery	Follow up calls at extra costs for attended home deliveries	●		●
	Employees’ basic needs, entitlement, and poverty of ground force	Lunch stipend			●
	Managing non-permanent and underperforming employees	Establishment of policies		●	●
		Mentoring programs, and		●	●
		Regular meetings to manage under performance			●

Such peculiarities are unique and differ from the practice of major e-grocery retailers, where orders are placed by customers rather than ground force personnel. However, the strategy of using ground force personnel by small and medium grocery e-retailers is crucial for scaling the use of the mobile application and for geographical penetration of the e-grocery market. Some have embarked on delivering workshops to help raise awareness of the potential and convenience of e-grocery shopping. "South African market does not understand e-groceries, which is a challenge because it means we need to educate customers. We are busy delivering workshops to help raise awareness". At worst, some customers' behaviors are not inclined to the electronic market and present extra telephone calls in case of attended home deliveries for mobile application retail and wholesale models, as captured by one of the CEO:

"Africans behave differently; for example, we had an order for a customer scheduled to be delivered on Monday but called him on a Friday to remind him that his order would be delivered on Monday morning. We checked if somebody would be home to collect, and this guy said, 'oh, I forgot about you guys; deliver on Friday instead.' So, imagine if we have not checked with him, we will sit with the stock for a week". [CEO of the mobile application wholesale model, SME #8].

Besides customer and market issues, small e-grocery retailers report issues relating to their employees' basic needs. There are issues of entitlement, and to some extent, the poverty of the ground force remains a reality in SA. Most young people taking up order entry jobs come for little or nothing. Therefore, e-grocery retailers need to offer them lunch. Thus, this adds extra costs to the township and rural model and is empathy at display.

"We have been working the whole day, and you guys are not giving us food. We are dealing with young people from the townships, and they think we should take care of them. Food is a big thing, and we said it is fine, R30 per head when they work a full day to help them find something to eat, it is a give and take […] First with staff, when we started, it was tough because we felt that most of the local youth feel entitled. They have entitlement issues, and most feel the government should give them things for free and do not understand the concept of working. Initially, people would come and go; we were finding it hard to get the right people". [CEO of the mobile application wholesale model, SME #3].

In addition, there are issues related to managing staff that underperforms, especially considering that most are temporary employees. Small e-grocery retailers have instituted policies, mentorship programs, and frequent meetings to help manage underperformance. The CEO of the mobile application brand model explained, "We have policies on everything from how to handle company information to company culture. We use the policies to manage when we have skills issues". The mentorship program enables job shadowing and identifies skills gaps to provide the necessary support, highlighting another cognitive element of e-grocery retailing for SMEs. "We pair them up with one of the most senior sales guys already, so together, s/he can pick up where the gaps are and areas to upskill." [CEO of the mobile application brand model, SME #1].

That is, SMEs are required to wear the hats of many business functions to make it in the trade. Hence, frequent meetings have been instrumental in giving the ground force a voice of concern, which helps small e-grocery retailers deliver feedback and training tailored to specific concerns.

"We also have a Tuesday meeting every morning where foot agents express their concerns or struggle. We use our meeting as an opportunity to skill and train our staff. Tuesday is a feedback session, and there is a complementary training session where we train and teach them marketing concepts, sales concepts, and customer profiling for them to succeed". [CEO of the mobile application wholesale model, SME #8].

## Discussions

The findings suggest that small firms are more likely to overcome most technology, organization, and environmental e-business and e-grocery adoption challenges if they are composed of teams with skills such as developers and software engineers. All eight case studies comprised teams with developers and software engineers, suggesting that e-grocery adoption is, to a great extent, related or influenced by the degree of small firms’ innovativeness and capabilities (or team-based adoption approach). Below is the discussion on the three theoretical constructs of TOE theory.

### TOE’s technological e-business/e-grocery adoption context

From the technological context, it was noticeable how the eight case studies limit their e-business and e-grocery challenges related to network infrastructure to the cost of data. Nevertheless, information network infrastructure extends beyond data to include, amongst others, mobile broadband, towers, optical fiber, ethernet, and cloud computing, which are all necessary for sustainable e-grocery operations [66, 67]. Indirectly, the small firms reveal, to a greater extent, the high penetration of telecommunication infrastructure in urban, township, and rural areas of SA and how such infrastructure has indirectly lowered the barriers for small firms. Hence, their description of network issues mainly centers on data costs. The findings support studies that have long acknowledged how technological infrastructure confers advantages to businesses within the context [67, 68]. The confirmation of expensive data costs by most small firms highlights the necessity of affordable data in advancing small firms or increasing entrepreneurship in Africa.

Regarding vehicle routing, open and free technologies such as Google Maps act as significant e-business/e-grocery adoption enablers, especially for small firms in the context of SA with high-income disparities and low-income countries, as previously observed by Faik and Walsham [69].

In addition, the case studies provide invaluable insight into the interplay of the TOE technological and organizational context. The teams’ dynamics and diverse composition of software engineers reveal the diverse digital and non-digital capabilities and skills (organizational context) that mitigate against the challenge of inventory systems and bespoke/information or data warehouse (technological context). The interplay of capabilities and skills from the TOE technological and organizational context expands on Awa et al.’s [53] and Yoon and George’s [54] critical determinants of compatibility, ease of use, and perceived advantage from its ‘superseding ideas’ contextualization, to include the cognitive dimension. At best, it reveals the nature of the cognitive dimension, a specification that builds support for and advocates for the cognitive dimensions of connective learning [70] and curiosity [35]. The cognitive dimension sheds light on some intangible resources that can be potential sources of a sustained competitive advantage.

### TOE’s organizational e-business/e-grocery adoption context

The e-business/e-grocery TOE’s organizational context breeds challenges related to the cost of hiring qualified staff, skills, profitable models, legal issues, trade finance, suitability to varied markets, managing roles and responsibilities, the freshness of products, availability, substitution, and supply and distribution.

The findings reveal a need for multilateral financial institutions to mobilize the growth of small e-grocery retailers. They build support, on the one hand, for the assertion by previous scholars that small firms struggle with access to finance [28], while on the other hand, the government subsidy exemplifies a public and private partnership to leverage the mobile applications to generate employment and propel small firms to become engines of economic growth. This insight confirms the relevance of including government support as the TOE’s crucial environmental factor to fuel small firms’ e-business adoption [13, 21].

Small e-grocery retailers reveal diverse profitable strategies that embody a shared value model. This is coopetition at display that provides alternative profitable models, according to Asdemir et al. [43]. However, concerns about losing control of the service level and e-grocery operations are bubbling under the surface. The concerns are consistent with the insight of those that tried to forge relationships with Uber drivers but retracted due to poor customer service. Moreover, the commission-based pay structure and appointment of independent contractors provide flexible entry to employment and market opportunities crucial for reducing unemployment.

Of greater importance, e-grocery mobile application models offer immersive digital experiences appropriate for varied market segments, which drive the demand for groceries. Small e-grocery retailers' practice removes some boundary conditions that have been perceived to limit the suitability of e-grocery to urban areas and the digitally savvy market segment, with low-income disparity, towards a model at the center of digital social inclusion. The removal of the boundary condition is in discord with scholars that limit e-grocery business to the urban context [71, 72].

Another area of customer expectation is the quality of fresh produce. Unlike major e-grocery retailers that own stock and contend with issues related to quality and freshness [33, 44], the small e-grocery retailers reveal insights that might provide them with unique sustained advantages in addressing the quality of fresh produce. An interesting perspective is how the demand for quality and fresh produce induces an undercurrent that supports small farmers and the local economy through a farm-to-fork strategy to meet e-grocery customers’ quality expectations.

From the three urban, township, and rural e-grocery mobile applications, the retail configured model had the highest unavailability of stock, the e-grocery mobile application brand/FMCG configured model reports no issue of availability, and The e-grocery mobile application wholesale configured model is the median of them all in terms of unavailability. The model-specific impact of unavailability changes the initial conception of availability and substitution from being a factor for the entire e-grocery operation [45, 46] to a factor that applies to the relative e-grocery model.

There appear to be differences in how efficiency and effectiveness are managed between big and small e-grocery retailers. For example, some small firms report no picking or delivery targets and instead prefer the single picking of products to increase the accuracy of orders. In contrast, big grocery retailers strive for efficiency and effectiveness of orders by managing pick and delivery targets [12]. It will be risky to conclude that one approach is better and safer to assert that different contexts and resources call for different priorities.

The overall impression gained from the study is that the context of small e-grocery retailers in terms of limited resources, township and rural markets, and the background of their ground force present issues and strategies peculiar to the specific context. For example, the poverty and background of their ground force call for the provision of lunch, and the digitally challenged market forced some to start providing workshops. These examples highlight some of the many peculiarities of the township and rural e-grocery operations specific to the context of SA. The interplay of the contexts establishes small grocery e-retailers as agents for addressing social and economic challenges such as poverty, unemployment, and inequality necessary for creating social and economic inclusion. Most specifically, it supports the literature that positions small businesses as the engine for accelerating economic growth [28, 73] and extends small businesses' position as the drivers of digital technologies for generating social impact.

### TOE’s environmental e-business/e-grocery adoption context

From the list of TOE’s environmental context e-business/e-grocery challenges, the road network infrastructure challenge appears in all the different e-grocery mobile application models. Furthermore, the riots that vandalize delivery vans in townships threaten the progress made by small e-grocery retailers. Against the bedrock of challenges, small firms appear innovative in mobile application development and survival strategies. While the majority mitigate by hiring staff with local knowledge to navigate around congestions, others are considering drones, e-cycles, and solar-powered containers in partnership with strategic national and private company experts from the world of navigation, CSIR, and solar panel suppliers, respectively.

Although not pronounced, a consolidation and observation of the findings suggest that key informal external stakeholders that emanate from the environmental context and that are critical and relevant for lowering the e-business/e-grocery adoption challenges include retail banks (payment packages such as speed points, e-wallets), ISP providers (data/internet), competitors’ stores (order fulfillment), township and rural households (act as the point of order entry and strategic partners), independent contractors (pickers, drivers, foot soldiers), government stakeholders (SETA and CSIR), digitally enabled urban markets (offices, executives and middle class), and most importantly, digitally challenged township and rural market (social grant holders, Kota traders, créches, bakeries, and societies, such as burial associations and stokvels. It remains, however, an interesting quest to determine how the Covid-19-induced work-from-home restructured the urban market segment.

Contrary to the assertion that competitors are expected to be a challenge for small firms' e-business/e-grocery adoption [13, 21], the study observed no emphasis on challenging competitors. While there could be many possible explanations, the study argues that this is mainly because the small e-grocery retailers in the context of the urban, township, and rural areas of SA embody coopetition. Competitors are considered strategic partners since they stock e-groceries from major grocery retailers. The latter argument lends support from Mkansi et al.’s [15] findings related to the profit sharing and support found between big and small e-grocery retailers and some of the fast-moving consumer goods (FMCG) companies.

All eight case studies reveal little, if any, financial support from the government, and their struggles in obtaining funding were listed in the analysis of challenges that pertain to the TOE organizational context. However, two case studies reveal some government-funded initiatives and stakeholders which significantly reduce the e-business/e-grocery organizational context challenges of labor. Indirectly, the support plays a crucial role in digital inclusion and bridging the digital divide because the small e-grocery deploys interns to the township and rural areas to process orders on behalf of the digitally challenged market segment. The findings solidify previous scholars’ assertions that small firms are the engines of economic growth [17, 74]. Beyond digital inclusion, the subsidy bridges skills gaps. Another support from a national institution reveals not only key government stakeholders related to minimizing e-grocery adoption challenges but also some of the key areas where the government can expand their support to transform townships and rural communities, empower small e-grocery retailers to increase jobs and employment, and help propel the efforts employed by the case studies in bridging the digital divide. These findings support Martin et al.’s [75] conclusion that online retailing lowers environmental impacts but with a fresh twist of shopping in the rural, township, and urban contexts.

A silent observation is how the small e-grocery retailers barely mentioned the telecommunication infrastructure as a challenge within the TOE environmental context. The observation supports and is consistent with the observation made in the analysis of the technological context challenges, which is that data is more of a problem than towers, broadband, and so forth are. Hence, the legal issues related to compliance are interconnected with those of the organizational context. As such, addressing the organizational legal issues and technological telecommunication infrastructure of e-business/e-grocery adoption, simultaneously address the environmental and legal framework and telecommunication infrastructural issues.

### TOE’s e-business/e-grocery adoption challenges revisited

**Fig. 4:**
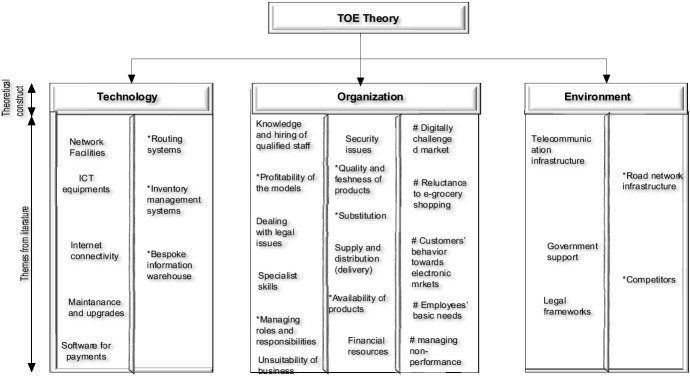
TOE e-business/e-grocery adoption challenges for SMEs (Revisited with emerging challenges). Notes: *Means e-grocery adoption context challenges; *#* means other emerging e-grocery challenges

The findings suggest emerging field-based adoption challenges faced by SME e-grocery mobile application practitioners in addition to the ones covered in the literature. Figure 4 presents a revision of Fig. 2 to include the emerging TOE’s e-business/e-grocery challenges stemming from this study. These emerging challenges fall within TOE organizational context and reveal issues and strategies peculiar to the specific context of small e-grocery retailers operating in the urban, township, and rural markets of South Africa. For example, the poverty and background of some of the SMEs' ground force call for the provision of lunch during the order processing phase. Furthermore, the digitally challenged market forced some SMEs to start providing workshops on the convenience of e-grocery shopping, revealing a unique aspect of the communication logistics elements required to penetrate the township and rural market.

## Conclusions

Small and medium e-grocery retailers operating in the urban, township, and rural areas of South Africa are swimming against the tide of double-edged e-business and e-grocery challenges and making headway into a complex digital and resource demanding market. This study applied the TOE framework to explore how small and medium e-grocery retailers in South Africa manage the double-layered e-business/e-grocery challenges and to determine their coping strategies.

Overall, the findings suggest that of the three theoretical constructs of TOE, the technological and environmental constructs pose the least barriers to e-grocery adoption in comparison to the organizational constructs in terms of the eight case studies. The organizational context barriers are mitigated mainly by orchestrating interconnected and interdependent technological and environmental context opportunities. The findings of mobile application e-grocery adoption challenges and coping strategies provide different layers of analysis with interesting insights contributing to theory and practice.

### Theoretical contributions

From the theory perspective, the organizational context of TOE poses more adoption challenges than the technological and environmental contexts. In terms of the logistics elements, the technological challenges of TOE affect order processing more than any other logistics elements. Hence, TOE’s organizational challenges are more prevalent in inventory, while TOE’s environmental challenges affect the transport logistics element more. Unitization and packaging are secondary logistics elements in mobile application e-grocery models and pose no challenges, as it is primary to major grocery retailers. The critical insight of how TOE’s theoretical context affects the logistics elements is crucial for reframing the critical determinants of e-business adoption.

Another emerging contribution to theory is how organizational challenges affect non-mobile e-grocery retailers and other mobile application e-grocery models differently. For example, organizational challenges such as the availability of products is not a challenge for the mobile applications FMCG/Brand model but affects the mobile retail application model and non-mobile e-grocery retailers, while it moderately affects the wholesale business model. Using foot agents to bridge digital gaps and reach the digitally challenged market is specific to mobile application wholesale and FMCG/brand models and the context of SA only. Looked at differently, this means mobile application e-grocers have a better reach and act as agents of digital transformation far more than non-mobile e-grocers and the big grocery retailers operating mainly in urban areas. The mobile application e-grocers have a better understanding of the base of the pyramid, which responds to the significant criticism of the theory’s inconclusiveness and unsuitability to the SMEs’ e-business adoption decisions.

The study further reveals that assessing mobile application e-grocery adoption and coping strategies in South Africa requires the disentangling of unique contextual issues, while the economic legacy offers superior explanatory power to e-business adoption theory. For instance, the customer-to-customer stock e-grocery exchange is observed in the mobile application wholesale model, mainly in the township and rural areas. Payment processing strategies such as electronic wallets, speed points, bank deposits, the Stokvel society model, and cash on delivery are observed in mobile applications only. The World Bank Report [76] and SAHRC Report [77] found South Africa to be the most unequal society in the entire world, where almost 64% of blacks and 41% of mixed or people of color live in poverty, while Indians and Whites make up only 6% and 1%, respectively, of the population. The unequal effects of the apartheid legacy are more evident in predominantly black areas such as townships and rural areas [78, 79]. Put differently, more than half of the population live in poverty and are predominantly digitally excluded, yet mobile application e-grocery adoption appears to thrive in those markets. Oduwole’s study [78] in achieving development goals in South Africa recognizes the structural complexity inherited from the systematic exclusion of blacks from participation in economic activities, one of the main factors linked to South Africa’s current triple challenge of poverty, inequality, and unemployment. The major implication to theory is that the sense of mobile application e-business/e-grocery adoption and coping strategies cannot be understood without understanding the systematic disparity of TOE’s theoretical context.

### Practical contributions

From the practice perspective, the coping strategies provide a blueprint for prospective mobile application e-grocery retailers and non-mobile e-grocery retailers. They further clarify the differences between the three mobile application e-grocery models and non-mobile grocery retailers. This is crucial for weighing trade-offs in adopting an e-grocery business. The insights for the practitioners’ adoption decision of a mobile application e-grocery business are outlined below and compared with the findings from non-mobile e-grocery adoption remedies discussed in the literature by previous scholars.

- Order processing challenges that involve foot agents have more impact on the mobile applications’ wholesale and FMCG/models but not on mobile application retail models.
- The pull approach that aggregates demand from townships, rural and urban areas reveal that inventory management issues could be lower for mobile application e-grocery retailers. This differs for non-mobile e-grocers operating a push approach, as shown in previous studies [12].
- Managing roles and responsibilities challenges, such as human errors, affect mobile and non-mobile application e-grocery retailers differently. Human errors in mobile application e-grocery retailers emanate from order processing and picking. However, as reported in previous studies [12], non-mobile e-grocery retailers primarily observe these in the picking process.
- Mobile retail and wholesale application models mitigate against availability by picking from other stores, a practice that is not evident and might not be feasible for non-mobile e-grocery retailers because, as reported in previous studies [12], they seek to increase their sales while managing their stock availability. Hence, mobile e-grocery retailers are more concerned with sales and profits and not much about stock availability.
- Major non-mobile application e-grocers serve as models for mobile application e-grocers in terms of legal compliance—their practices and legal compliance lower the e-business adoption barriers associated with e-business.
- Organizational e-grocery challenges, such as the cost of substitution, affect non-mobile e-grocery retailers more than the mobile applications models. Indeed when the desirable items are unavailable, close substitutes are used by non-mobile e-grocery retailers to satisfy customers and sometimes at higher replacement costs [12]. This study shows that the stock availability issue is less prominent to mobile application e-grocery retailers.
- Financing options like family borrowing and personal savings are entry modes for mobile application e-grocery models.
- Exploring intermodal transportation, future technologies such as drones, solar-powered multi-purpose centers, and community alliance strategies are similar for mobile application e-grocery and non-mobile e-grocery retailers (for instance, Amazon uses parcel lockers in stations and many other places). Previous studies have pointed out how non-mobile application e-grocery retailers cope with issues regarding managing competitive delivery windows against demand and costs or balancing traffic congestion on the road and on-time deliveries [12]. The difference is that mobile applications add value to townships and rural areas with no economic structure (apartheid context).

The study has limitations. The insight offers empirical evidence of how mobile application e-grocery distribution models pan out in developing economies, especially how SMEs address e-grocery operations. Notably, the findings are limited to the South African context and the case studies under study. While the study offers a great understanding of how mobile application e-grocery retailers address theoretical condensed and emerging adoption challenges, it does not demonstrate the relationship between the development of mobile technologies and the opportunities and barriers to the development of e-business models in the e-grocery sector. Accounting for the latter, both in relation to theoretical and practical considerations, there are other implications for future studies that have emanated from our research. Our findings suggest that small firms are more likely to overcome most of the technology, organization, and environmental e-business and e-grocery challenges if they are composed of teams with skills, such as developers and software engineers. Future studies can perform a longitudinal study to explore how the performance of those small grocery e-retailers with prior retailing experience, coopeting partners, and software engineering teams facilitate firm growth and sustainable advantage against those without these premises. Other studies can further investigate how team composition renders specific ‘perceived critical determinants’ of e-business adoption less critical.

## Supplementary Information

Below is the link to the electronic supplementary material.

Supplementary file1 (DOCX 801 KB)

Supplementary file2 (DOCX 28 KB)
