# Supplementary material for: Mobile application e-grocery retail adoption challenges and coping strategies: a South African small and medium enterprises’ perspective
Source: Electron Commer Res. 2023 Apr 25:1–46. Online ahead of print. doi: 10.1007/s10660-023-09698-1 (PMC10127987; doi:10.1007/s10660-023-09698-1)
Supplement: Supplementary file 1 — Supplementary file1 (DOCX 801 KB) [file 10660_2023_9698_MOESM1_ESM.docx]

Appendix 1

Appendix 2

Appendix 3
