# Supplementary material for: Mobile application e-grocery retail adoption challenges and coping strategies: a South African small and medium enterprises’ perspective
Source: Electron Commer Res. 2023 Apr 25:1–46. Online ahead of print. doi: 10.1007/s10660-023-09698-1 (PMC10127987; doi:10.1007/s10660-023-09698-1)
Supplement: Supplementary file 2 — Supplementary file2 (DOCX 28 KB) [file 10660_2023_9698_MOESM2_ESM.docx]

Summary of TOE’s e-business/e-grocery adoption challenges and coping strategies

| **TOE construct** | **Challenges** | **Coping strategies** | **Mobile Application Models** | | |
| --- | --- | --- | --- | --- | --- |
|  |  |  | **Mobile App retail** | **Mobile App brand/ FMCG** | **Mobile App warehouse** |
| **Technological** | Network facilities, ICT equipment, and maintenance and upgrades | Interface with organizational construct strategies of specialist skills, and team diversity that consists of software engineers and developers. | ● | ● | ● |
|  | Internet connectivity | Negotiating with media center to be a master distributer of data. |  |  | ● |
|  |  | Use built-in features to monitor and optimize data usage. | ● | ● |  |
|  |  | Accept and pay the cost. | ● |  |  |
|  | Routing systems | Exploit capabilities of Google Maps. | ● | ● | ● |
|  |  | Foot agents captures coordinates during order process, which later feeds to delivery addresses. |  | ● | ● |
|  | Software for Payments | Use a combination of flexible payment methods such as: electronic wallets, electronic funds transfer (EFT), speed point, cash on delivery, bank deposit, credit, and PayPal. | ● | ● | ● |
|  |  | Stokvel society model |  |  | ● |
|  | Inventory management systems | Develop built-in features that offer visibility of stocks |  | ● | ● |
|  |  | Substitution features on the mobile app (interface with organizational context strategy for managing substitutions). | ● |  |  |
|  | Bespoke information or data warehouse | Tracing and tracking of demand by location. | ● | ● | ● |
|  |  | Understand stock consumption patterns. | ● | ● | ● |
|  |  | Profiling of customers. |  | ● | ● |
|  |  | Link mobile application to a web-based platform which uses machine learning to understand stock consumption patterns that need to be dropped to different townships |  | ● |  |
| **Organizational** | Knowledge and hiring of qualified staff | Own internal skills as developers. | ● | ● | ● |
|  |  | Orchestration of innovative teams with complementary skills. | ● | ● | ● |
|  |  | Leverage sustainable labor approaches (e.g., subsidized learnership or internships). |  |  | ● |
|  |  | Commission strategy. |  | ● | ● |
|  | Security issues | Use of cash on delivery options with or without target limits and e-wallets. | ● | ● | ● |
|  |  | Carry minimal amount of stock and/or cash. | ● | ● | ● |
|  |  | Use of a pull model. | ● | ● | ● |
|  | Quality and freshness of products | Shoppers’ training programs to place quality at the center of their picking activities. | ● | ● | ● |
|  |  | Embedding quality specification features on mobile applications that provide customers with freshness specification options at the point of order. | ● |  | ● |
|  |  | Use of farm-to-fork approach. | ● | ● |  |
|  | Availability of products | Seek customers’ alternative options through direct calls. | ● |  | ● |
|  |  | Search for missing products anywhere for the promised price and delivery delay. | ● |  |  |
|  |  | Customer-to-customer loan in exchange for a credit note. |  |  | ● |
|  |  | Seeking availability from local supermarkets through direct calls. | ● |  |  |
|  |  | Built-in features in the mobile applications to issue alternatives for product with high risk of unavailability. | ● |  | ● |
|  | Substitution | Real-time suggestion to customers in case of products unavailability. | ● |  |  |
|  |  | Use a proactive method where customer recommend alternatives for those products that have high probability of unavailability. |  |  | ● |
|  | Managing roles and responsibilities: human errors | Built-in features that enable adding notes to the order. |  |  | ● |
|  |  | Use of bar codes to confirm order accuracy. | ● |  |  |
|  |  | Fostering single-picking over multiple picking. | ● |  |  |
|  |  | Bear the brunt of re-delivery and the associated costs. | ● |  |  |
|  | Special skills | Offer competitive rates. | ● | ● | ● |
|  |  | Do not contend with PDB license | ● | ● | ● |
|  | Financial resources | Loans from family and friends. | ● | ● | ● |
|  |  | Savings. | ● | ● | ● |
|  | Profitability models | Geo-pricing strategy. |  |  | ● |
|  |  | Uber partnership. | ● |  |  |
|  |  | Mini-distribution centers (DCs) (Spaza shops partnership) Warehousing, |  | ● | ● |
|  |  | Shared value student model. | ● |  |  |
| **Emerging Organisational** | Digitally challenged market (unable to place orders on the app). | Deploy foot agents. |  | ● | ● |
|  | Reluctance in terms of e-grocery adoption (trust issues). | Raise awareness of the potential and convenience of e-grocery shopping. |  |  | ● |
|  | Customers’ behavior toward electronic market, i.e. unavailability of customers during delivery. | Follow up calls at extra costs for attended home deliveries. | ● |  | ● |
|  | Employees’ basic needs, entitlement, and poverty of ground force | Lunch stipend |  |  | ● |
|  | Managing non-permanent and underperforming employees | Establishment of policies |  | ● | ● |
|  |  | Mentoring programs, and |  | ● | ● |
|  |  | Regular meetings to manage under performance |  |  | ● |
| **Environment** | Road network infrastructure | Use of an intermodal transport system involving vans and other means (e.g., bicycles, wheelbarrows, etc.). |  |  | ● |
|  |  | Protect against riots by purchasing insurance. | ● |  |  |
|  |  | Working with local drivers accustomed to township driving patterns. | ● | ● | ● |
|  |  | Exploring future technologies such as drones, solar powered multi-purpose center and community strategic alliances. |  |  | ● |
|  |  | Exploring e-cycling program and partnership with the Council for Scientific and Industrial Research (CSIR). |  |  | ● |
|  |  | Pick from the nearest mall or shopping center. | ● |  |  |
|  | Dealing with legal issues | Consultation with lawyers. | ● |  | ● |
|  |  | Observation of big grocery e-retailers’ practices. | ● |  | ● |
|  |  | Awareness of policies and current affairs. | ● | ● | ● |
|  |  | Use of frozen bags with prescribed temperatures for frozen products. | ● |  |  |
|  |  | Offer credit in exchange for another purchase. |  |  | ● |
|  |  | Tries to align with Labor Act to some extent. |  |  | ● |
